# Non-coding RNA gene families in the genomes of anopheline mosquitoes

**DOI:** 10.1186/1471-2164-15-1038

**Published:** 2014-11-28

**Authors:** Vicky Dritsou, Elena Deligianni, Emmanuel Dialynas, James Allen, Nikos Poulakakis, Christos Louis, Dan Lawson, Pantelis Topalis

**Affiliations:** Centre for Functional Genomics, University of Perugia, Perugia, Italy; Institute of Molecular Biology and Biotechnology, FORTH, Heraklion, Greece; Department of Biology, University of Crete, Heraklion, Greece; Natural History Museum of Crete, University of Crete, Heraklion, Greece; European Bioinformatics Institute, Hinxton, UK

**Keywords:** Anopheles, Genome evolution, microRNA, ncRNA, Ribosomal genes, Small nuclear RNA, Small nucleolar RNA, tRNA, Whole Genome Sequencing

## Abstract

**Background:**

Only a small fraction of the mosquito species of the genus Anopheles are able to transmit malaria, one of the biggest killer diseases of poverty, which is mostly prevalent in the tropics. This diversity has genetic, yet unknown, causes. In a further attempt to contribute to the elucidation of these variances, the international “Anopheles Genomes Cluster Consortium” project (a.k.a. “16 *Anopheles* genomes project”) was established, aiming at a comprehensive genomic analysis of several anopheline species, most of which are malaria vectors. In the frame of the international consortium carrying out this project our team studied the genes encoding families of non-coding RNAs (ncRNAs), concentrating on four classes: microRNA (miRNA), ribosomal RNA (rRNA), small nuclear RNA (snRNA), and in particular small nucleolar RNA (snoRNA) and, finally, transfer RNA (tRNA).

**Results:**

Our analysis was carried out using, exclusively, computational approaches, and evaluating both the primary NGS reads as well as the respective genome assemblies produced by the consortium and stored in VectorBase; moreover, the results of RNAseq surveys in cases in which these were available and meaningful were also accessed in order to obtain supplementary data, as were “pre-genomic era” sequence data stored in nucleic acid databases. The investigation included the identification and analysis, in most species studied, of ncRNA genes belonging to several families, as well as the analysis of the evolutionary relations of some of those genes in cross-comparisons to other members of the genus Anopheles.

**Conclusions:**

Our study led to the identification of members of these gene families in the majority of twenty different anopheline taxa. A set of tools for the study of the evolution and molecular biology of important disease vectors has, thus, been obtained.

**Electronic supplementary material:**

The online version of this article (doi:10.1186/1471-2164-15-1038) contains supplementary material, which is available to authorized users.

## Background

Although it is a historic fact that control of malaria, as well as of most vector-borne diseases, has only been achieved through the control of the transmitting arthropod vectors, mosquito-related research has seriously lagged behind *Plasmodium*-related research for some time. It is only since the formation of the McArthur Foundation Network on Vector Biology [1] that a significant thrust was given to this study area. This increased research involvement soon led to the acquisition of the whole genome sequence of *Anopheles gambiae*[2], the most important African malaria vector; among insects, this was the second completed genome after that of *Drosophila melanogaster*[3]. The wealth of pertinent biological information available today for mosquitoes, combined with the tremendous increase of the power of genomics, has now made it easier to address projects that a few years ago would have been considered completely impractical. It was therefore natural that a consortium of more than 100 scientists was recently formed with the goal of sequencing and studying several genomic aspects of 16 anopheline species, many of which constitute important malaria vectors in several areas of the world [4]. In addition to obvious tasks such as genome assembly and general annotation, the project included the study of genes and gene families that were either important for the understanding of the vectors’ biology, or that constituted target objects for inclusion in molecular approaches aiming at controlling disease transmission. The latter group included, for example, genes involved in chemosensation, detoxification and insecticide resistance, as well as genes whose products are found in the saliva of the mosquitoes, while the former included, among others, repetitive elements and non-coding RNA genes. Our team assumed responsibility for the study of the latter genes.

Several distinct RNA species have been detected during the last years in addition to the “classical” three RNA classes, mRNA [5, 6], rRNA [7] and tRNA [8] that helped describe the central dogma of Molecular Biology established as such later [9]. The vast majority of those RNA species is known to not encode polypeptides; they are, therefore, collectively branded as non-coding RNAs or ncRNA [10]. The population of ncRNAs in any organism is made out of several distinct families of RNA species that are, usually, not related to each other, although the individual members of a given family, in addition to a common function, often share sequence and/or structure characteristics.

In the initial part of our involvement in the project, which we describe here, we chose to focus on four of these ncRNA families: the rRNA genes, including the chromosomally unlinked 5S rRNA genes, the small nucleolar RNA genes (snoRNA), the nuclear tRNA genes, and the miRNAs. Here we describe the analysis of these genes in their genomic context using a pure computational approach and expand this to include a study on the sequence evolution of the ribosomal genes. Although the “*Anopheles* Genomes Cluster Consortium”, of which we are part, initially focused on the analysis of 16 different genomes, we expanded the study with the analysis of additional anopheline genomes for some gene families described here, due to their availability in the meantime. Therefore, in several cases we report the identification of ncRNA genes in up to 20 different genomes.

## Methods

### Species and assemblies

The assembled genomes of the following anopheline species were used in this analysis. For those taxa for which more than one assembly was available, the one used is indicated in parentheses following the name of the species: A. albimanus*, A. arabiensis*, A. atroparvus*, A. christyi, A. coluzzii, A. culicifacies, A. darlingi, A. dirus*, A. epiroticus, A. farauti* (AfarF1), A. funestus* (AfunF1)*, A. gambiae* (AgamP4 and AgamS), *A. maculatus, A. melas* (AmelC1), A. merus* (AmerM1), A. minimus*, A. quadriannulatus*, A. sinensis* (AsinS1), A. stephensi* (AsteS1)*.* RNAseq data [11] were used, when appropriate/available, for the species that are marked, above, with an asterisk. We note that a) some of these genomes (*A. darlingi* and *A. stephensi*) were not part of the species originally chosen for the “16 *Anopheles* genomes project” and b) for some ncRNA families some genomes did not yield any significant results, probably due to miss-assemblies, and are therefore excluded from the corresponding sections. Sequencing, assembly and annotation are described by [11]. All assemblies and sequences are publically available at VectorBase [12].

### Identification of rRNA genes

To identify the ribosomal gene repeat, assemblies available through VectorBase were queried with different sequences using its BLAST [13] server. This was initially done using as queries, individually, the respective homologous *D. melanogaster* sequences encoding the 5.8S, 18S and 28S genes [14]. To potentially close gaps that were present in almost every repeat in all genomes analyzed, raw reads stored at the Sequence Read Archive/SRA repository [15] were blasted using as queries, this time, the sequences previously identified with the BLAST searches performed for each species; contigs and consensus sequences were then manually assembled to the extent that this was possible. Finally, sequences present in Genbank were also compared to the sequences identified as above. In all cases in which Genbank contained ribosomal sequences for which we had not identified counterparts, those were retrieved and included in the output. The output should be considered a consensus sequence of the rDNA segment analyzed.

The same strategy used for the ribosomal repeat was also used for the isolation of the 5S rRNA gene, again starting with the *D. melanogaster* 5S gene [16] as a query. BLAST searches of RNAseq experiments stored at the SRA repository were likewise used to determine the exact size of ribosomal transcripts for the 5S genes where available.

### Phylogenetic analysis

A total of 5.145 bp of concatenated DNA sequences (5S rRNA, 18S rRNA, 28SrRNA, mitochondrial 16SrRNA and COI) retrieved from 17 *Anopheles* species were phylogenetically analyzed (see Additional file 1). *Drosophila melanogaster* sequences [14] was used as an outgroup taxon.

DNA sequences were aligned using MAFFT v.6 [17] with auto (for COI) and Q-INS-i (for rRNA) strategies, removing ambiguous and poorly aligned regions. Each gene fragment was aligned separately. The best-fit model of DNA substitution was chosen for each gene fragment with jModelTest v. 2.1.5 [18], according to the Akaike Information Criteria (AIC). The analysis was run under 5 substitution schemes, base frequencies estimation (+F), gamma shape (+G) and invariable sites (+I) estimation, which makes a total of 40 models. The models including both G and I were ignored [19]. The above parameters concluded in that K80, GTR + G, TrN + G, GTR + G, and GTR + G were the best fit models for the five fragments of genes (5S rRNA, 18S rRNA, 28SrRNA, 16SrRNA, and COI, respectively).

Bayesian Inference (BI), Maximum Likelihood (ML), and Neighbor-Joining analyses were conducted in MrBayes (v3.2.2) [20], RAxML (v. 7.2.7) [21] and MEGA (v. 6.0.6) [22], respectively. In all analyses nucleotides were used as discrete, unordered characters.

BI analysis was performed with four runs for 10^7^ generations and eight chains, using the K80, GTR + G, TrN + G GTR + G, and GTR + G models of evolution for 5S rRNA, 18S rRNA, 28SrRNA, 16SrRNA, and COI, respectively, based on the results of the AIC. The current tree was saved to file every 100 generations. This generated an output of 10^5^ trees for every run. The performance of the runs was visualized using Tracer v1.6 [23]. The first 25*10^3^ trees (25%) were discarded as “burn-in” and a majority rule consensus tree was calculated from the remaining trees. The posterior probabilities were calculated as the percentage of samples recovering a clade.

The ML analysis was performed under the GTRGAMMA model (General Time Reversible model of nucleotide substitution under the Γ model of rate heterogeneity). To ensure that the inferred ML tree was not a local optimum 200 ML searches for each dataset were conducted. The Robinson-Foulds symmetric distance was employed to assess the topological similarity between these trees [24]. The confidence of the branches of the best ML tree was further assessed based on 1000 rapid bootstrap replicates (under the GTRCAT model) (for more details see [21]).

### Identification of tRNA genes

To identify tRNA genes in the assemblies of the different anopheline genomes we screened the assembled sequences with tRNAScan-SE [25]. To detect genes that potentially escaped the first search, we also used full genomic alignments of all scaffolds from all anophelines analyzed produced by Robert Waterhouse (MIT and University of Geneva) as input to the RNAZ 2.0 suite [26]. Any putative positive prediction was then BLASTed to the Rfam database [27, 28].

### Identification of miRNA genes

For the identification of miRNAs a dual approach was chosen, *ab initio* predictions and similarity searches. Two different pieces of software were used for *ab initio* predictions, HHMMIR [29] and MiRPara [30]. Both treat genomic sequences as RNA molecules and predict their secondary structure. Next, they check the thermodynamic stability of those structures and they classify them as potential miRNA genes or not. HHMMIR and MiRPara differ in the classification algorithm, the first one using a Hidden Markov Model (HMM) whereas the second depends on a Support Vector Machine (SVM). A second difference is that HHMMIR was trained to predict (positive set) animal miRNA genes in general, while the positive set for MiRPara included miRNA genes from *Anopheles gambiae, Aedes aegypti, Culex quinquefasciatus* and *Drosophila* melanogaster, annotated as such in miRbase, v20 [31, 32]. In both cases, each scaffold of every genome was analyzed separately in order to parallelize the process. Predictions with a confidence score lower than 80% were discarded. The remaining output (usually in the order of tens or hundreds of thousands) was kept for further filtering. The second computational strategy consisted of a similarity-based approach. Here, we used two ways to identify miRNA genes. The first consisted of querying, in BLAST searches, with known miRNAs from *Aedes aegypti*, *Culex quinquefasciatus*, *A. gambiae* and other invertebrate organisms stored at the corresponding section of RefSeq at NCBI, and present in the miRNA database miRBase v20. The second similarity scheme was to map, with zero mismatches allowed, to the anopheline genomic assemblies the mature miRNAs presently available in miRBase v20. The regions identified were then checked for the presence of miRNA genes by combining the mapping results with those of the other lines of evidence. Finally, we used the RNAz pipeline [26] to identify genomic regions in the assemblies that could contain non-coding RNA genes. We used genomic alignments for all the genomes available (kindly provided by Robert Waterhouse). Both MiRPara and HHMIR “chopped” every scaffold in segments of 521 bps in length with an overlap of 176 bps. Since RNAz analyzes alignments of a maximum of 6 sequences, multiple samples were taken and the presence of thermodynamically stable, non-coding RNAs was calculated. Any prediction with a confidence score lower than 90% was discarded. The presence of miRNA genes was then detected by BLASTing positive hits to the Rfam database. Rfam was last updated on August 2012; to potentially increase the number of putative miRNA genes, we BLASTed our hits versus the RefSeq-RNA database (downloaded 10 July 2014).

The results of the five lines of evidence were combined. miRNA genes predicted/identified by at least two different pipelines were considered to represent *bona fide* genes if the length of the predicted hit was greater than 70 bps. Also, since positive hits from genomic predictors or high scoring segments produced by BLAST usually do not start at the very same base, overlapping hits or hits that start within an area of 40 bps were considered as representing the same gene. Hits on opposite strands, even when in the same region, were kept in the final set. Several PERL scripts were written throughout this project. They were used as wrappers to facilitate the analysis of each scaffold separately in the local HPC cluster, or to combine, filter and compare the results compiling the final gene set.

### Identification of small nucleolar RNAs (snoRNAs)

Prediction of C/D box snoRNAs was performed using snoScan [33]; candidate sequences returned with an initial score of >20 were retained. They were then examined “manually” and they were classified based on the computed possibility of the presence of a stem: no stem, possible stem, terminal stem and strong stem. SnoReport [34] was also used initially for an independent prediction of snoRNAs but, even using a probability score of pSVM >0.99, snoRNA genes were overpredicted, i.e. many more genes were predicted than what was expected from other organisms; SnoReport was, thus, not used further.

### Identification of small nuclear RNAs: snRNAs

We used the RNAz pipeline [25] to identify genomic regions in the assemblies that contained genes putatively coding for snRNAs.

## Results and discussion

### The rDNA gene repeat

Although among the very first genes to be isolated and described in metazoa (*e.g.*[35]), rRNA genes remain difficult study entities in the genomic era. Because genomes usually contain hundreds of copies of both the main rDNA repeat (that include the 5.8S, 18S and 28S genes) and the small, unlinked 5S rDNA repeats, their precise assembly is extremely tedious and, often, impossible. This is aggravated by polymorphisms, among others due to the frequent interruption of the genes in the main repeats by repetitive elements such as the ones found early on in *D. melanogaster* ([36, 37]) and other insects [38], including *A. gambiae*[39]; these insertions usually, but not exclusively, interrupt the 28S gene. Finally, often more than one locus containing rDNA genes exist in a given genome, typically in both sex chromosomes. In *A. gambiae*, however, rDNA is found on the *X* chromosome [40] although the possibility that some repeats are also localized on the Y chromosome cannot be excluded [41].

Examples of the difficulties encountered in the genomics of rDNA can be seen in the genome assemblies stored in VectorBase. The AfunF1 assembly of *A. funestus*, for example, only contains one 18S and two 28S genes, all in a segment of ~50 kb, arranged in a non-canonical way. Moreover, in addition to *A. gambiae* only 2 and 7 anopheline species contain annotations for the 28S and 18S ribosomal genes, respectively in VectorBase. Finally, although 14 anophelines are listed as containing (few) copies of the 5S rRNA gene, these are wrongly annotated throughout as 5.8S RNA genes, though with shorter lengths. It should be noted that miss-annotations are not restricted to the assemblies in VectorBase. For example, the 3′ end of 5.8S gene of *A. atroparvus* (Genbank accession # AY050640) has been annotated to a nucleotide corresponding, in reality, to base pair #97; it should be noted, though, that the sequence beyond this nucleotide contains several polymorphisms when compared to the one determined in the present study. As a result of this and other similar inconsistencies, we chose to initially disregard any previous annotations available, until we could verify them with the data acquired in the present analysis.

The results of the rDNA repeat analysis are summarized in Table 1, while all sequences identified the new anopheline assemblies [11] or database mining are reported in the Additional file 2.

**Table 1:** **The rDNA repeat in 17 anophelines**

Species	ETS “L”	ETS “S”	18S “L”	18S “S”	18S “C”	ITS1 “L”	ITS1 “S”	ITS1 “C”	5.8S	5.8S “S”	5.8S “C”
*Albimanus*	1538	*L78065*	1977	t.s.	Full	242	*L78065*	Full	160	t.s.	Full
*Arabiensis*	0		1981	t.s.	Full	346	*DQ287772*	Full	160	t.s.	Full
*Atroparvus*	0		1965	AM072973 [42]	Full	0			144	t.s.	part
*Christyi*	0		583	t.s.	Part	0			155	t.s.	Part
*Culicifacies*	0		0			345	*EU244872*	Full	160	t.s.	Full
*Dirus*	0		823	AF417779 [43]	Part	0			160	t.s.	Full
*Epiroticus*	0		1786	t.s.	Full (?)	0			160	t.s.	Full
*Farauti*	0		2046	AF121054 [44]	Full	1194	EF042721 [15]	Full	160	t.s.	Full
*Funestus*	0		1818	t.s.	Full	0			160	t.s.	Full
*Gambiae*	0		2015	AM157179 [2]	Full	344	AAAB01006374 [5]	Full	160	t.s.	Full
*Maculatus*	0		1950	*AF440198*	Full	0			160	t.s.	Full
*Melas*	0		0			0			160	t.s.	Full
*Merus*	0		1518	t.s.	Part	0			160	t.s.	Full
*Minimus*	0		1059	t.s.	Part	0			160	t.s.	Full
*Quadriannulatus*	0		1045	t.s.	Part	0			160	t.s.	Full
*Sinensis*	0		1795	t.s.	Full (?)	0			143	t.s.	Part
*Stephensi*	0		1903	t.s.	Full (?)	345	*EU244871*	Full	160	t.s.	Full
**Species**	**ITS2 “L”**	**ITS2 “S”**	**ITS2 “C”**	**28S “L”**	**28S “S”**	**28S “C”**	**NTS “L”**				
*Albimanus*	244	*L78065*	Full	4022	t.s.	Full	0				
*Arabiensis*	434	*DQ287772*	Full	1550	t.s., U10138, [45]	Part	1398				
*Atroparvus*	308	*AY050640*	Full	0			0				
*Christyi*	419	*GQ870324*	Full	0			0				
*Culicifacies*	368	*AY427754*	Full	553	t.s.	Part	0				
*Dirus*	506	*DQ629915*	Full	537	AF41781 [43]	Part	0				
*Epiroticus*	572	*AF469855*	Full	0			0				
*Farauti*	564	EF042721 [46]	Full	546	AF417815 [43]	Part	0				
*Funestus*	724	*JN994135*	Full	3445	t.s.	Part	0				
*Gambiae*	434	*X67157*	Full	4021	t.s.	Full	1733				
*Maculatus*	363	*AY803346*	Full	391	*AY120851*	Part	0				
*Melas*	437	*GQ870314*	Full	440	AF087512 [47]	Part	1118				
*Merus*	437	*GQ870313*	Full	440	AF087514 [47]	Part	862				
*minimus*	381	*JN975457*	Full	811	t.s.	Part	0				
*Quadriannulatus*	465	*JN994146*	Full	440	AQU10137 [47]	Part	1540				
*Sinensis*	469	GU384695 [46]	Full	4166	t.s.	Full	2896				
*Stephensi*	466	*AY157316*	Full	4096	t.s.	Full	0				

The Table summarizes the data for the rDNA segments ETS, 18S, ITS1, 5.8S, ITS2, 28S and NTS. “L” includes the length of the sequence assembled for the corresponding DNA segment in base pairs, “S” the source of the sequence and “C” its completeness.

Full: full length of genomic segment available; part.: only partial sequence of the genomic segment available; t.s.: this study; number in square brackets: published reference; alphanumeric number: Genbank/EMBL accession number. Accession numbers in italics refer to sequences obtained from public databases (Genbank/EMBL) for which no published reference is indicated. The question marks refer to the uncertainty as to the completeness of the sequence (see Results).

#### The 18S RNA genes

In all organisms examined, the 18S genes are the first of the ribosomal RNAs to be synthesized when Polymerase I initiates transcription at a promoter located at the end of the Non Transcribed Spacer (NTS, also called intergenic Spacer - IGS) of the rDNA repeat unit, giving rise to a large precursor RNA molecule that includes all rRNA species. This is then processed yielding the individual rRNA molecules. In *D. melanogaster* the primary transcript is led by a 864 nucleotides long segment called External Transcribed Spacer (ETS) [14], later to be “chopped off” during maturation. The summary of our analysis is presented in Table 1 and Additional file 2. While in the fruit fly the 18S rRNA is 1995 nucleotides long [14], we found that, in those species in which a complete 18S gene was identified, its length ranges from 1786 bp (*A. epiroticus*) to 2046 bp (*A. farauti*) (see Table 1). It should be noted, though, that we cannot ascertain that the 18S sequences of *A. epiroticus*, *A. sinensis* and *A. stephensi* are complete, possibly missing a few bases at their 3′ end. By BLASTing the available SRA sequences and the assemblies present at VectorBase, as well as individual entries in Genbank, we managed to identify and assemble the complete 18S rDNA sequences in 10 of the 17 *anophelines* analyzed (including the three aforementioned species)*.* In 6 more species, only a partial assembly was achieved: at least 1000 bp were identified in 4 taxa, but only 600 bp in two of them. Finally, we were unable to identify unambiguously any of the 18S sequences for *A. culicifacies* and *A. melas* using the available resources.

#### The 5.8S RNA genes

Insect 5.8S rRNAs, like their homologues in most living organisms, are encoded by a gene that is located in the ribosomal gene repeat between the 18S and the 28S genes [35] separating the long internal non-coding segment into the two internal transcribed spacers ITS1 and ITS2. An interesting feature of the 5.8S gene in many insects [48] is the fact that it is split into two parts: a longer one of about 120 bp in length, conventionally still called 5.8S, and a shorter 30 bp long one named 2S [49]. In *D. melanogaster* the latter is separated by a 28 bp long transcribed segment [14] that is later removed giving rise to two distinct small RNA species that interact to perform the same function of the “canonical” contiguous 5.8S ribosomal RNA, that interacts with specific ribosomal proteins during translation [50]. Although “split” 5.8S rRNA genes have been described in Drosophila, they should not be considered a common characteristic of diptera. As a matter of fact, in six culicidae examined to date, none was identified in which the mature 5.8S rRNA gene was made out of the two individual, processed species [48, 51].

**Figure 1:**
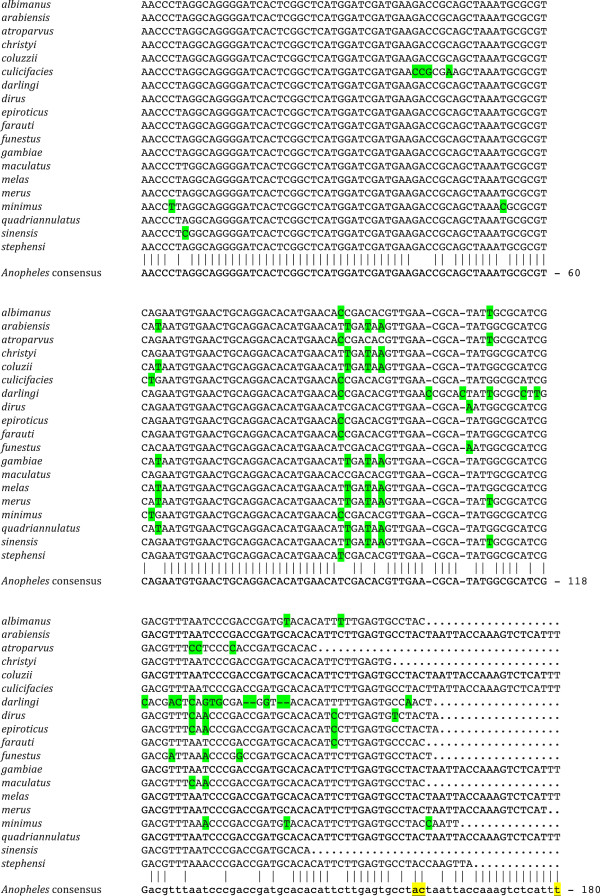
**Alignments of genes encoding the 5.8S ribosomal RNA.** The species examined are shown at the left before the sequences. Nucleotides highlighted in green differ from those found at the corresponding position in the consensus sequence. Dashes indicate gaps introduced to improve the alignment, dots to sequence that was not identified. Capitalized letters in the consensus sequence indicate the extent of the 5.8S RNA in *D. melanogaster*. The three underlined nucleotides highlighted in yellow in the *Anopheles* consensus sequence point to the terminal nucleotides of the three RNA species identified through the analysis of RNAseq experiments. The base numbering refers to the consensus sequence.

BLAST searches of both the assembled genomes and the SRA collections of reads that were generated in this project, allowed us to identify the 5S rRNA gene homologues in all species examined; in 16 out of 19 we were able to assemble a segment coding for the full 5.8S species. Initially we used the *D. melanogaster* sequence as a query, and then switched to that of *A. gambiae* once this was unambiguously identified. The sequences shown in Figure 1 show alignments of consensus sequences for each individual species; an overall consensus sequence for all anophelines was assembled from those of the 19 species examined. We stress here that the consensus of each individual taxon is based on the BLAST searches of the primary sequence reads; the output was obviously biased towards sequences that were more similar to the BLAST query; they are therefore not to be considered as “statistical representatives” of all reads present in the SRA database. Not unexpectedly, as seen in Figure 1 a very high degree of sequence conservation is apparent which, overall, ranges from 100% in comparisons between members of the *A. gambiae* s.l. species complex, to ~89% when the sequence of *A. darlingi* is compared to the consensus sequence determined from all species examined (average >96%). It should also be noted that most of the polymorphisms seen in *A. darlingi* are clustered towards the 3′ end of the mature 5.8S molecule, following the pattern detected for the overall comparison: we have determined a total of 42 polymorphic sites, of which 7 (17%) are found in the 5′-most 60 nucleotides, 13 (31%) in the next 60 bases and the remaining ones over the last segment of the gene.

As described for the only anopheline that had previously been studied, *A. pseudopunctipennis*[52] as well as one unnamed species [48], our sequence analysis and comparisons to the 5.8S gene of *D. melanogaster* suggest that the 5.8S gene is not interrupted in any of the 19 species examined: Similarity to the fruit fly gene drops after nucleotide 122 (not shown), while it is maintained throughout the anophelines. Analysis of BLAST searches performed on transcribed sequences in several species suggest that two main classes of 5.8S rRNA are present in the species analyzed, with a length of 159 and 160 nucleotides, although reads corresponding to molecules that are 20 nucleotides longer can be detected at a ratio of about 1:10 compared to the shorter ones (see Figure 1).

#### The 28S RNA genes

The results of the 28S RNA gene analysis are also summarized in Table 1 and Additional file 2. To identify the 28S RNA genes, a similar procedure to that chosen for the 18S genes was followed. It was first guided by the 28S gene of the fruitfly, which is 3945 bp long [14]. Unfortunately, the procedure used for identifying these sequences by BLASTing the assembled genomes as well as the SRA collections of reads was not as successful as with the 18S rRNA and the 5.8 rRNA genes. More precisely, only in 4 out of 17 species analyzed (*A. albimanus*, *A. gambiae*, *A. sinensis*, *A. stephensi*) were we able to identify a complete gene and in one species (*A. funestus*) a large part of the 28S gene (Table 1). In 9 other species we recognized shorter segments, their length ranging from 391 bp to 1550 bp, while no sufficient results were retrieved for the 3 remaining species (namely *A. atroparvus, A. christyi* and *A. epiroticus*). The assemblies in VectorBase did not contain complete genes. We assume that the failure to retrieve complete sequences in most genomes examined is due, as mentioned earlier, to the presence of interruptions in the contiguity of the 28S genes from the insertion of non-ribosomal DNA into them. We decided to only list here (Additional file 2) genes for which an unequivocal assembly can be presented.

#### The spacers

Spacer regions of the rDNA repeat were early on identified as excellent tools for the identification of cryptic taxa in anophelines [53]. This was an important technical development given that mosquito (and other arthropod) vectors are often members of species complexes whose members are difficult to distinguish. For the purpose of vector control it is important to be able to differentiate easily between vector and non-vector members of those complexes [47]. We therefore invested effort in identifying and classifying spacer regions. It should be noted that this was an extremely difficult and, often, unsuccessful exercise; the reason was that the spacer regions have undergone a substantial sequence diversification throughout evolution. We therefore stress that even in the case of the ITS2, the spacer separating the 5.8S and the 28S genes, which we identified in all species studied through corresponding entries in Genbank, we cannot absolutely ascertain the validity of the database records. BLASTing both SRAs and VectorBase assemblies often yielded results that could not be validated: discontinuities resulted in “jumps” from the ribosomal locus into other, non-identified regions of the genome (i.e. sequences initially considered to be contiguous were eventually found to be derived from -non-linked chromosomal segments). This is particularly important, because the ITS2 spacer is the one that is mostly used for phylogenetic studies [47]. The Genbank entries for the ITS2 region have lengths ranging from 244 bp to 724 bp (Table 1).

The hunt for ITS1 (i.e. the region between the 18S and the 5.8S genes), as well as for the NTS and ETS, were even less successful. In no cases were we able to identify the DNA sequences by analyzing the new data obtained through the “16 *Anopheles* genomes project”; since querying with available sequences never yielded any positive results, we attempted “walks”, but these equally led to the failure of identifying contiguous segments. Other than blaming potential miss-assemblies, pointing to the reasons for that failure would be sheer speculation (*e.g.* insertions of repeated sequences, and others) Thus, Table 1 only lists sequences for those spacers that had been earlier deposited in databases or published (Genbank Accession numbers in Table 1 and [2, 45, 46, 54–56]).

Finally, the only anopheline species for which a complete intergenic spacer (NTS plus ETS) has ever been described is *A. sinensis*, its total length being 2896 bp. We compared the sequence of this segment to the partial sequences of the spacers available for five members of the *gambiae* complex (not shown). Only when several gaps were introduced along the segments closest to the 3′ end of the 28S gene could some regions of, possibly, insignificant similarity be observed; however, when the NTS segments of the five members of the *gambiae* complex were compared to each other, extensive similarities were evident. This was particularly true, again, for the segments closest to the 3′ end of the 28S gene.

### The 5S rRNA genes

The eukaryotic 5S rRNAs, with lengths ranging from 115 to 125 nucleotides, are not related to the prokaryotic RNA species of the same name (see [57]). In *D. melanogaster* the mature 5S rRNA molecule is 120 bases long, stemming from a primary transcript of 135 bases that is post-transcriptionally shortened from its 3′ end [57–59]. About 100 copies of the gene encoding this rRNA are clustered at the cytogenetic locus 56 F1-56 F2; they are arranged as tandem repeats of a unit length of about 375 bp consisting of the mature RNA-coding segment and a spacer DNA [16, 60]. In all insects analyzed so far sizes of the mature RNA are conserved, while the length of the overall repeat usually represents a multiple of a nucleosome length (core plus spacer). In addition to the functional constrains on the actual 5S RNA molecule stemming from its involvement in the translation machinery through direct interactions with the 18S molecule (see [61]) the repeat length may be dictated by the potential phasing of nucleosomes [62], participating in the regulation of the transcription by RNA Polymerase III [63].

**Figure 2:**
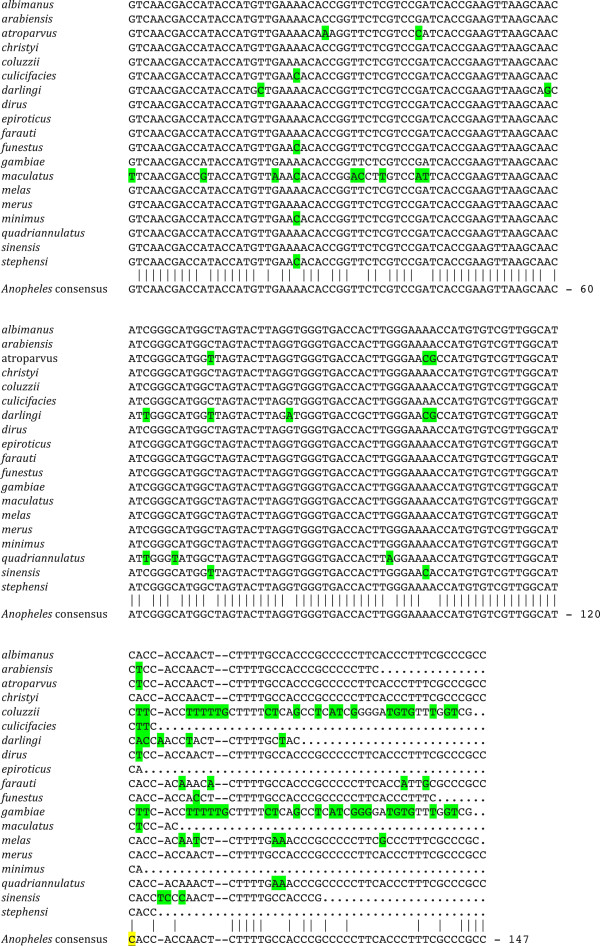
**Alignments of genes encoding the 5S ribosomal RNA.** The species examined are shown at the left before the sequences. Nucleotides highlighted in green differ from those found at the corresponding position in the consensus sequence. Dashes refer to indels, periods to sequences that were not identified. The nucleotide corresponding to the most frequently used 3′ terminus (see section on 5S RNA) is underlined and highlighted in yellow in the *Anopheles* consensus sequence. The base numbering refers to the consensus sequence, excluding the dashes.

Little, so far, is known about 5S RNA genes in mosquitoes and no entry describing the complete or partial sequence of this RNA class is available in nucleic acid databases. To isolate the 5S rRNA gene we again used the *D. melanogaster* mature molecule to query the different genomes in BLAST searches of the SRAs. We succeeded in assembling the individual sequence of the mature 5S RNA genes in 19 genomes. A “consensus” sequence was also assembled for all species analyzed (Figure 2).

Starting from the 5′ end of the fruit fly gene, we detected a high degree of similarity across all species studied (Figure 2). The sequences are absolutely collinear for 124 bases, with one extra nucleotide appearing at position +125 in *A. darlingi* and *A. sinensis*. We interpret this “insertion” to perhaps represent the first base of the intergenic spacer, although the mature RNA could well be shorter: searches of RNA sequences are inconclusive and need additional experimental information to be interpreted. The majority of the BLAST hits of RNA-seq SRA reads showed molecules that extended to nucleotide #121, although we could detect both shorter, up to #115, and longer ones, up to #139 (not shown). These “aberrant” molecules could represent errors in either transcription or maturation of the 5S RNAs. Nevertheless, we think that a short U-rich sequence (CTTTT) downstream of the presumed mature RNA, which is equidistant to a similar sequence in *D.* melanogaster, could represent the canonical signal for the end of transcription [64–66]. Interestingly, the segments between nucleotide 121 and the CTTTT sequence are highly polymorphic, including two indels of one and two bases in four of the species studied, as mentioned above.

We assembled a consensus sequence for for all 19 species examined (Figure 2). We stress here that this consensus is based on the BLAST searches of the primary sequence reads and not the assembled genomic sequences. The degree of similarity over the first 121 base pairs, across all anopheline species studied, is ~85% (cumulative number of nucleotides that are different from the “consensus”) although, of course, the similarity between any two species is, indeed, much higher than that.

Looking at the sequence of the first 121 nucleotides (Figure 2), the species whose 5S rDNA sequence differs most from the consensus of the 19 genomes analyzed are *A. maculatus* (8 nucleotides, all between bases 11 and 42), *A. darlingi* (7 nucleotides, all between bases 19 and 102) and *A. atroparvus* (6 nucleotides, all between bases 1 and 102). *A. quadriannulatus* has three bases different from the consensus, *A. sinensis* has 2, while only one base pair differentiates *minimus*, *stephensi* and *culicifacies* from the consensus. The most common polymorphism affects nucleotide #24 (five times), while none of the others is found in more than three species. Finally, comparing *D. melanogaster* to the anopheline consensus sequence, one notices 32 differences between them scattered over the first 120 nucleotides, or a conservation of 73% (not shown).

In the *A. gambiae* AgamP4 assembly available at VectorBase, sequences similar to the 5S ribosomal RNA are found at the cytogenetic locus 23C; like in the fruit fly, the genes are unlinked from the remaining rRNA genes. The AgamP4 assembly indicates a series of ~500 bp long tandem repeats, which are wrongly annotated as being 5.8 rRNA genes of a length of 115 bp each. Although longer than that described for the fruit fly, the overall length of the repeat is consistent with the theory of nucleosome phasing, whereby three nucleosomes of ~167 bp could be localized on each of the repeats, instead of two as in *D. melanogaster*. This would be a situation similar to *Xenopus laevis* where the repeat of the 5S gene is equal to four nucleosomes [67].

### Phylogenetic analysis

**Figure 3:**
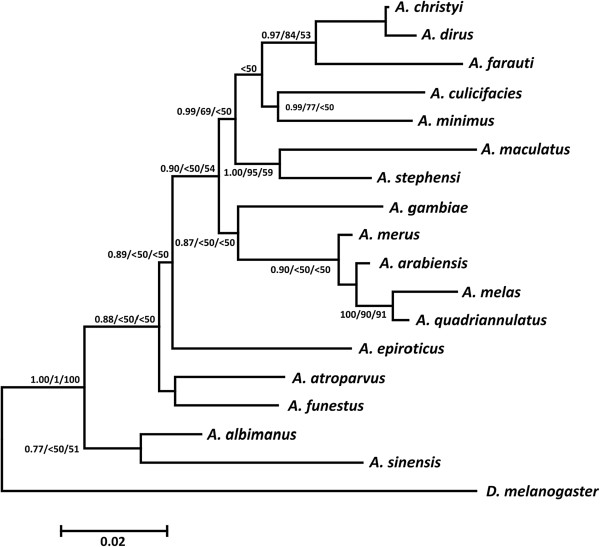
**Bayesian Inference tree inferred by the concatenated dataset.** The numbers on the branches indicate posterior probabilities and bootstrap supports (BI/ML/NJ).

The absence of complete rDNA sequences for some of the species combined with the fact that the alignment procedure led to remove a large part of them as unaligned (the alignment was ambiguous) made us decide to restrict our analysis to a concatenated sequence that included a portion of the nuclear ribosomal sequences produced in this study and segments of the mitochondrial 16S rDNA gene and the COI genes. Our aim was to identify the origin of these ribosomal sequences and also to evaluate the produced phylogenetic relationships based on the previously published data. A total of 5,145 base pairs (bp) for all loci (5S rRNA: 170 bp, 18S rRNA: 1332 bp, 28SrRNA: 826 bp, 16SrRNA: 1338 bp, and COI: 1479 bp) were analyzed for 18 taxa (17 ingroup taxa of the genus *Anopheles* and one outgroup taxon *D. melanogaster*). The ingroup alignment contained 921 variable and 395 parsimony informative sites, while when the outgroup taxon were included they were raised to 1229 and 472, respectively. Maximum Likelihood (-lnL = 16177.91), Bayesian Inference (-lnL = 16201.02) and Neighbor Joining analyses of the concatenated data produced similar topologies (see Figure 3). Although without very good statistical support (posterior probabilities in BI and bootstrap values in ML and NJ), the produced tree revealed several groups of species. One of them is the *Anopheles gambiae* complex that includes *A. gambiae* that branched off first, *A. merus, A. arabiensis, A. melas*, and *A. quadriannulatus*, which is in agreement with previous published analyses [45]. The other major group comprises 7 species in which *A. maculatus* seems to be sister taxon to *A. stephensi* (1.00/95/59) and *A. culicifacies* to *A. minimus* (0.99/77/<50).

### The snoRNA genes

snoRNAs are a family of small nucleolar RNA species that are involved in post-transcriptional modification of other ncRNA classes, primarily rRNAs, but possibly also tRNAs and snRNAs (see [68]). Two main classes are known, the C/D box snoRNAs, which are primarily associated with methylation of target RNAs, and the H/ACA box snoRNAs that are mostly involved in pseudouridilation processes [69]. To date, no published reports exist describing any of these RNA classes in insects other than Drosophila. We therefore initiated a search for snoRNAs in the genomes of the “16 *Anopheles* genomes project”.

snoRNA genes are difficult to identify *de novo* in large genomes. For the prediction of C/D box snoRNA we concentrated on the usage of snoScan [25], a computational method that predicts the genes based on target recognition, in combination with SnoReport, a genome-wide approach [33] that uses a combination of RNA secondary structure prediction and machine learning based on known snoRNAs; unfortunately, we found that SnoReport “overpredicted” snoRNA genes in all genomes analyzed (the numbers for all species were 4–6 times higher than those expected from comparisons to other organisms). Taking a conservative approach, we report here the results of the analysis of snoScan, although we can provide the SnoReport data on demand (see Additional file 3).

snoScan requires the input of “target” RNA sequences; in our cases the sequences were “extracted” from the consensi of the 18S and 28S rDNA determined. In cases in which the rDNA sequences available were considered to be too short, we used instead the corresponding nucleic acids from the closest neighbor in evolutionary terms [11]. Also, in the case of the two *A. gambiae* strains and *A. coluzzii,* the same consensus sequence of *A. gambiae* was used (see Table 2).

**Table 2:** **snoRNA genes in 19 anopheline taxa**

Species	18S target from:	Length of 18S target	No stem	Possible stem	Terminal stem	Strong stem	Total	Genes/kb
*Albimanus*		1977	30	17	62	19	128	6.5
*Arabiensis*		1981	20	10	51	21	102	5.1
*Atroparvus*		1965	15	8	40	7	70	3.6
*Christyi*		583	3	1	5	1	10	1.7
*Coluzzii*	*Gambiae*	2015	20	11	38	25	94	4.7
*Culicifacies*	*Funestus*	1817	14	4	19	10	47	2.6
*Dirus*		594	8	2	11	2	23	3.9
*Epiroticus*		1786	12	12	19	12	55	3.1
*Farauti*		2046	26	19	46	11	102	5
*Funestus*		1817	14	9	23	8	54	3
*Gambiae* PEST		2015	31	13	94	22	160	7.9
*Gambiae* S		2015	24	13	43	22	102	5.1
*Maculatus*		1950	9	5	29	9	52	2.7
*Melas*	*Gambiae*	2015	13	11	41	23	88	4.4
*Merus*		1518	19	11	43	17	90	5.9
*Minimus*		1059	7	6	13	4	30	2.8
*Quadrianulatus*		1045	10	7	20	12	49	4.7
*Sinensis*		1795	14	5	29	6	54	3
*Stephensi*		1903	30	13	46	10	99	5.2
**Species**	**28S target from:**	**Length of 28S target**	**No stem**	**Possible stem**	**Terminal stem**	**Strong stem**	**Total**	**Genes/kb**
*Albimanus*		4022	80	43	158	32	320	8
*Arabiensis*		1110	17	8	21	8	55	5
*Atroparvus*	*Sinensis*	4069	40	35	76	22	174	4.3
*Christyi*		524	4	3	9	3	19	3.6
*Coluzzii*	*Gambiae*	3440	63	30	82	36	220	6.4
*Culicifacies*		560	7	6	6	1	20	3.6
*Dirus*	*Farauti*	546	8	6	25	12	53	9.7
*Epiroticus*	*Christyi*	524	4	4	12	8	28	5.3
*Farauti*		546	9	3	12	5	29	5.3
*Funestus*		3445	43	21	53	16	134	3.9
*Gambiae* PEST		3440	75	39	126	52	300	8.7
*Gambiae S*		3440	66	35	98	29	236	6.9
*Maculatus*		351	2	2	7	0	11	3.1
*Melas*		440	4	1	6	1	12	2.7
*Merus*		440	5	2	10	2	19	4.3
*Minimus*		811	7	5	15	2	31	3.8
*Quadrianulatus*		440	3	2	8	1	14	3.2
*Sinensis*		4096	42	15	61	12	129	3.1
*Stephensi*		3801	56	21	91	21	189	5
*Sinensis*	*Sinensis*	4096	42	15	61	12	129	3.1
*Stephensi SDA*	*Stephensi*	3801	54	20	92	21	189	5.0

The table lists both the taxa examined and those whose sequences were used as targets if different (see Results). The numbers refer to the individual candidate genes containing the different snoRNA structures as well as their total and the number of putative genes per 100 bp of target sequence used. The length of the target sequences is also indicated.

Unexpectedly, although we aimed at predicting both C/D box and H/ACA box snoRNAs with snoScan, only sequences corresponding to C/D box-containing snoRNA genes could be identified. We assume that this is due to the C/D box snoRNAs’ well-conserved motifs and the 10-21 nt complementary guide sequences that lie between the motifs [70], which enabled the successful computational screen. In contrast, H/ACA guide snoRNAs are shorter and have less well-conserved primary sequence motifs, therefore being harder to identify.

Table 2 lists the results of the analysis for predicted C/D box snoRNAs in the Anopheles taxa studied. The lowest numbers of snoRNA genes targeting 18S RNA predicted were 23 (*A. dirus*) and 29 (*A. minimus*), while the highest were 168 (*A. gambiae* PEST strain) and 130 (*A. albimanus).* If only the highest stringency predictions are considered, the number of genes drops to numbers lower than 23 for all taxa examined. The number of predictions per 100 bases of target RNA ranges from 2.6 to 8.3, the latter number referring to the usage of the *A. gambiae* PEST strain assembly. It should be emphasized that that particular genome is represented by pre-NGS whole genome sequence; this “technical” difference might be the reason for higher predictions; we could speculatively state that this might be due to better quality sequencing.

The total number of putative snoRNA genes targeting 28S RNA can be seen in Table 2. Here, not unexpectedly, the taxa with the lowest numbers of predicted genes are the ones for which a shorter target was provided. The number of predictions per 100 nucleotides of target are not significantly different among the different taxa.

In *Drosophila melanogaster* 98 C/D box snoRNAs have been annotated in the current (May 9, 2014) release of FlyBase [71], although it is possible that more exist. 64 of them have 28S rRNA as a target, 30 target 18S rRNA while the remaining four are thought to be involved in the methylation of U6 snRNA (2x), U2 snRNA and 5.8S rRNA. Interestingly, but not unexpectedly, we detect in the case of anophelines a high degree of similarity between the snoRNA genes in closer-related species. Within the members of the *gambiae* complex, more than 30% of the genes are 100% identical (not shown). A few snoRNAs are also shared between more distantly related species (*e.g. A. epiroticus* has one gene that has an identical sequence with one in *A. farauti*).

### The tRNA genes

In all organisms examined so far tRNA genes constitute the largest gene family; membership ranges from a few hundreds to several thousands (see [72] in different species examined. The extremely conserved “cloverleaf structure” combined with the preserved localization of functional sequence features on it [72, 73] have made it easy, early on, to devise strategies for the computational identification of these genes from a large variety of organisms. Although there is a rough correlation between the number of tRNA genes and the “complexity” of an organism, the actual number of tRNA genes [74] is not directly proportional to the genome size as can be exemplified when one looks at diptera. While *D. melanogaster*, with a genome size of ~125 Mb contains between 590 and 950 tRNA genes [75]. *Aedes aegypti* (genome size: ~1.3 Gb) contains 906 and *A. gambiae* 441 with a ~270 Mb long genome [76]. The latter numbers include 111 and 1 pseudogenes, respectively.

The findings of this analysis are reported in Additional file 4 and summarized in Table 3. Gene numbers in different species should be considered as approximate since, in spite of the solid methodology used, gene assemblies rarely represent the reality, even of the actual specimens sequenced. This can be exemplified by the greatly differing number of tRNA genes determined for *A. gambiae*, when the assemblies of two different strains present in VectorBase were analyzed (see Table 3). While we found 389 genes in the AgamS1 assembly of the S form Pimperena strain, the PEST strain assembly (AgamP4) yielded 464 genes, i.e. a number that is 19.3% higher. Interestingly, using the same software as we used in the present study, Behura & Severson [76] in an earlier gene set of the AgamP4 assembly identified 24 fewer tRNA genes (see Table 3). Also, these authors identified one pseudogene versus 11 in the present study, and no tRNA for Selenocysteine, while we identified one such gene. The number of tRNA genes computed ranged from 125 for *A. minimus* to 464 in the *A. gambiae* PEST strain. The average number is 331.4 tRNA genes per genome, or 1.5 tRNA genes per Mb of genomic DNA. As can also be seen in Table 3, the only species examined that is unusual is the Asian mosquito *A. maculatus*. Here, the number of tRNA genes identified is 125, or ~37.7% of the average number and, the number of genes per Mb is also substantially lower than the average (0.9). One should note that this species also has the second-smallest genome assembled. The low number of genes in the genome of *A. maculatus* also leads to statistically significant but, probably, biologically less relevant differences in the relative abundance of isoacceptors for some amino acids (see Table 3).

**Table 3:** **tRNA genes identified in 19 anopheline species**

	- > Aliphatic										- > S-containing				P	Σ	M	F
Anopheline species	Gly	%	Val	%	Ala	%	Leu	%	Ile	%	Cys	%	Met	%				
*Albimanus*	17	5.6	15	4.9	28	9.2	20	6.5	13	4.2	5	1.6	15	4.9	0	306	170.5	1.8
*Arabiensis*	22	6.1	28	7.8	25	7.0	22	6.1	16	4.5	5	1.4	18	5.0	6	359	246.6	1.5
*Atroparvus*	21	6.2	26	7.7	24	7.1	23	6.8	12	3.6	5	1.5	17	5.0	1	337	224.3	1.5
*Christyi*	18	6.0	20	6.7	20	6.7	17	5.7	11	3.7	5	1.7	14	4.7	8	300	172.7	1.7
*Coluzzii*	24	6.3	22	5.8	27	7.1	22	5.8	16	4.2	5	1.3	18	4.7	10	379	224.5	1.7
*Culicifacies*	21	7.3	23	8.0	18	6.3	19	6.6	10	3.5	6	2.1	13	4.5	118	286	203.0	1.4
*Darlingi*	10	4.4	12	5.3	16	7.0	14	6.1	9	3.9	3	1.3	11	4.8	0	228	134.7	1.7
*Dirus*	23	6.7	23	6.7	26	7.5	23	6.7	12	3.5	5	1.4	16	4.6	3	345	216.3	1.6
*Epiroticus*	18	5.6	22	6.9	24	7.5	19	5.9	13	4.1	5	1.6	16	5.0	67	320	223.5	1.4
*Farauti*	22	6.2	25	7.1	22	6.2	23	6.5	14	4.0	5	1.4	17	4.8	0	354	181.0	1.9
*Funestus*	17	5.9	24	8.4	18	6.3	15	5.2	10	3.5	6	2.1	12	4.2	0	286	225.2	1.3
*Maculatus*	6	4.8	10	7.9	8	6.3	9	7.1	***3***	***2.4***	4	***3.2***	6	4.8	5	126	141.9	0.9
*Melas*	20	5.7	23	6.6	26	7.4	24	6.9	16	4.6	5	1.4	17	4.9	15	349	227.4	1.5
*Merus*	22	6.3	25	7.1	25	7.1	22	6.3	14	4.0	5	1.4	18	5.1	8	351	251.8	1.4
*Minimus*	18	5.8	***42***	***13.5***	18	5.8	23	7.4	11	3.5	5	1.6	18	5.8	134	312	201.8	1.5
*Gambiae* AgamP4	29	6.2	***65***	***14.0***	28	6.0	28	6.0	15	3.2	5	1.1	21	4.5	11	465	278.0	1.7
*Gambiae* AgamP4*	25	5.7	***64***	***14.5***	28	6.4	26	5.9	15	3.4	5	1.1	19	4.3	1	440	278.0	1.6
*Gambiae* AgamS	27	6.9	26	6.7	29	7.5	28	7.2	14	3.6	5	1.3	21	5.4	13	389	236.4	1.6
*Quadrianulatus*	22	6.2	25	7.0	25	7.0	22	6.2	14	3.9	4	1.1	17	4.8	7	357	283.8	1.3
*Sinensis*	20	5.6	26	7.2	23	6.4	26	7.2	14	3.9	6	1.7	18	5.0	2	360	241.4	1.5
*Stephensi*	18	5.6	27	8.3	19	5.9	17	5.2	12	3.7	5	1.5	16	4.9	83	324	225.4	1.4
Average aa per species	20.0	6.0	27.3	8.0	22.7	6.8	21.0	6.4	12.6	3.8	5.0	1.6	16.1	4.8	23.4	331.4	218.5	1.5
	**- > Acidic & their amide cont. aa**								**- > Basic**									
**Anopheline species**	**Asp**	**%**	**Asn**	**%**	**Glu**	**%**	**Gln**	**%**	**His**	**%**	**Arg**	**%**	**Lys**	**%**				
*Albimanus*	14	4.6	10	3.3	23	3.3	12	3.9	15	4.9	19	6.2	22	7.2				
*Arabiensis*	18	5.0	11	3.1	25	3.1	15	4.2	20	5.6	21	5.8	24	6.7				
*Atroparvus*	18	5.3	10	3.0	22	3.0	13	3.9	12	3.6	21	6.2	23	6.8				
*Christyi*	16	5.3	12	4.0	26	4.0	13	4.3	16	5.3	20	6.7	22	7.3				
*Coluzzii*	18	4.7	11	2.9	25	2.9	16	4.2	20	5.3	24	6.3	31	8.2				
*Culicifacies*	12	4.2	8	2.8	22	2.8	13	4.5	11	3.8	18	6.3	18	6.3				
*Darlingi*	12	5.3	5	2.2	16	2.2	8	3.5	16	7.0	14	6.1	17	7.5				
*Dirus*	16	4.6	10	2.9	26	2.9	15	4.3	13	3.8	24	7.0	24	7.0				
*Epiroticus*	16	5.0	9	2.8	23	2.8	14	4.4	15	4.7	20	6.3	23	7.2				
*Farauti*	18	5.1	11	3.1	27	3.1	15	4.2	12	3.4	21	5.9	25	7.1				
*Funestus*	13	4.5	7	2.4	22	2.4	13	4.5	13	4.5	19	6.6	19	6.6				
*Maculatus*	5	4.0	3	2.4	6	2.4	5	4.0	8	6.3	13	***10.3***	8	6.3				
*Melas*	18	5.2	13	3.7	22	3.7	14	4.0	14	4.0	23	6.6	25	7.2				
*Merus*	19	5.4	11	3.1	24	3.1	15	4.3	16	4.6	21	6.0	25	7.1				
*Minimus*	13	4.2	8	2.6	19	2.6	13	4.2	12	3.8	18	5.8	18	5.8				
*Gambiae* AgamP4	26	5.6	14	3.0	28	3.0	15	3.2	23	4.9	24	5.2	29	6.2				
*Gambiae* AgamP4*	26	5.9	12	2.7	27	2.7	15	3.4	23	5.2	23	5.2	25	5.7				
*Gambiae* AgamS	18	4.6	11	2.8	26	2.8	16	4.1	17	4.4	22	5.7	25	6.4				
*Quadrianulatus*	18	5.0	11	3.1	25	3.1	15	4.2	19	5.3	21	5.9	26	7.3				
*Sinensis*	22	6.1	9	2.5	29	2.5	13	3.6	19	5.3	22	6.1	27	7.5				
*Stephensi*	14	4.3	9	2.8	31	2.8	15	4.6	14	4.3	19	5.9	18	5.6				
Average aa per species	16.7	5.0	9.8	2.9	23.5	2.9	13.5	4.1	15.6	4.8	20.3	6.3	22.6	6.8				
	**- > Aromatic**						**- > OH-containing**				**- > Cyclic**		**- > Seleno.-cont.**					
**Anopheline species**	**Phe**	**%**	**Tyr**	**%**	**Trp**	**%**	**Ser**	**%**	**Thr**	**%**	**Pro**	**%**	**Sec**	**%**				
*Albimanus*	9	2.9	14	4.6	6	2.0	19	6.2	15	4.9	14	4.6	1	0.3				
*Arabiensis*	9	2.5	21	5.8	6	1.7	20	5.6	15	4.2	18	5.0	0					
*Atroparvus*	9	2.7	17	5.0	6	1.8	18	5.3	16	4.7	23	6.8	1	0.3				
*Christyi*	9	3.0	9	3.0	5	1.7	17	5.7	15	5.0	15	5.0	0					
*Coluzzii*	9	2.4	24	6.3	6	1.6	21	5.5	15	4.0	25	6.6	0					
*Culicifacies*	9	3.1	11	3.8	5	1.7	18	6.3	16	5.6	13	4.5	2	0.7				
*Darlingi*	8	3.5	16	7.0	6	2.6	12	5.3	9	3.9	13	5.7	1	0.4				
*Dirus*	11	3.2	18	5.2	6	1.7	19	5.5	16	4.6	18	5.2	1	0.3				
*Epiroticus*	9	2.8	19	5.9	5	1.6	18	5.6	15	4.7	17	5.3	0					
*Farauti*	11	3.1	21	5.9	6	1.7	21	5.9	17	4.8	18	5.1	3	0.8				
*Funestus*	9	3.1	17	5.9	5	1.7	17	5.9	15	5.2	15	5.2	0					
*Maculatus*	***2***	***1.6***	***6***	4.8	2	1.6	8	6.3	***10***	***7.9***	***3***	***2.4***	1	0.8				
*Melas*	9	2.6	20	5.7	6	1.7	20	5.7	17	4.9	17	4.9	0					
*Merus*	9	2.6	22	6.3	6	1.7	19	5.4	15	4.3	18	5.1						
*Minimus*	9	2.9	14	4.5	5	1.6	16	5.1	15	4.8	17	5.4	0					
*Gambiae* AgamP4	13	2.8	23	4.9	8	1.7	23	4.9	15	3.2	32	6.9	1	0.2				
*Gambiae* AgamP4*	9	2.0	22	5.0	7	1.6	22	5.0	15	3.4	32	7.3	0					
*Gambiae* AgamS	9	2.3	25	6.4	7	1.8	21	5.4	15	3.9	27	6.9	0					
*Quadrianulatus*	9	2.5	24	6.7	6	1.7	19	5.3	16	4.5	19	5.3	0					
*Sinensis*	9	2.5	21	5.8	6	1.7	18	5.0	15	4.2	17	4.7	0					
*Stephensi*	10	3.1	17	5.2	5	1.5	19	5.9	14	4.3	23	7.1	2	0.6				
Average aa per species	9.0	2.7	18.1	5.4	5.7	1.7	18.3	5.6	14.8	4.6	18.8	5.5	0.7	0.5				

The columns under the amino acid name show the number of tRNA isoacceptor genes determined for each amino acid indicated, while neighboring columns show the respective percentage of the total number of tRNA genes for that isoacceptor in that particular species. Cells with characters in bold/italic font show numbers that differ significantly for that particular tRNA from the other species. The taxon indicated as *gambiae* AgamP4* refers to the numbers obtained by Behura and Severson (Behura and Severson 2011; see Results). The four additional columns at the right-hand side of the first part of the table show additional data such as (from left to right) the number of pseudogenes detected (PG), the total number of tRNA genes in the species (Σ), the genome size based on the assemblies (Mb) and the number of tRNA genes per Mb of genomic sequence (F).

Finally, we should point to the fact that the genomes of both the *A. gambiae* PEST strain as well as *A. minimus* were found to contain a number of valine tRNA genes that is significantly higher (about double) than of the other species. Surprisingly enough, this is neither the case for the Pimperena strain, nor the remaining 5 members of the complex, including *A. coluzzii*, a taxon that was recently elevated to a species [77], having previously been considered to be a “molecular form” of *A. gambiae* s.s. [78]. We cannot suggest an explanation for this finding other than, potentially, an artifact due to the genome assembly.

### The miRNA genes

miRNAs are a class of small RNA molecules that have been found to play a crucial role in the regulation of gene expression in metazoans and plants [79]. miRNAs are transcribed as precursor molecules that undergo processing via mechanisms that have been studied [80]. The so-called pre-miRNA hairpins, are the first discrete molecules of a length of 50–70 nucleotides to appear, and these are exported to the cytoplasm where they are processed to the 22–23 nucleotides long miRNA molecules. There, miRNAs pair to mRNA molecules leading to posttranscriptional silencing of protein-coding genes [81]. miRNAs may play crucial roles in vector-borne diseases: A number of miRNA molecules (and the genes that encode them) been recently reported in mosquitoes [82–85] and the biological role attributed to them also includes a hypothetical involvement in the regulation of pathogen-vector interactions such as *Plasmodium berghei-A. gambiae*[86] as well as processes more closely associated with the pathogenesis process [85].

The identification of *bona fide* miRNA molecules usually requires a complicated pipeline consisting of a computational search, the detection of the molecules among reads of small RNA sequencing surveys and, finally, the functional identification of the miRNA-target interactions. Given the fact that the present analysis was part of the “16 *Anopheles* genomes project”, we had to concentrate on the first of the three approaches. We decided to take a rather conservative attitude in terms of calling the actual miRNA genes identified and used stringent criteria calling positives. We included in our analysis both *ab initio* prediction software as well as homology searches of three kinds. Only when two of these pointed to a potential candidate sequence as being a miRNA would we accept the finding. The results of this analysis are summarized in Table 4 while Additional file 5 lists all putative miRNA genes identified.

**Table 4:** **miRNA genes discovered**

	miRNA predictions - this study	Previously annotated as miRNA (miRBAse)	Putative new miRNAs: Refseq ncRNA	miRNA - already in VB	Common genes - this study/VB	% Common -this study/VB
*Albimanus*	96	89	7	67	53	55.2
*Arabiensis*	95	88	7	83	58	61.1
*Atroparvus*	96	93	3	53	38	39.6
*Christyi*	93	85	8	69	60	64.5
*Coluzzii*	43	43	0	0	0	0
*Culicifacies*	71	71	0	75	39	54.9
*Darlingi*	55	55	0	57	18	32.7
*Dirus*	110	110	0	61	37	33.6
*Epiroticus*	85	79	6	73	53	62.4
*Farauti*	108	99	9	62	48	44.4
*Funestus*	113	104	9	66	48	42.5
*Gambiae-PEST*	63	58	5	172	21	33.3
*Gambiae-S*	61	61	0	0	0	0
*Maculatus*	40	40	0	53	35	87.5
*Melas*	65	65	0	82	38	58.5
*Merus*	118	107	11	86	58	49.2
*Minimus*	48	48	0	65	35	72.9
*Quadriannulatus*	97	92	5	75	55	56.7
*Sinensis*	115	105	10	76	50	43.5
*Stephensi*	111	107	4	65	45	40.5

The table lists the number of genes discovered and compares them to the list of miRNA genes annotated in miRBase, VB or the RFAM and RefSeq databases.

Our pipeline yielded a number of putative miRNA genes. In case that this was possible, we annotated all miRNA genes identified with either the miRBase name or the name of the ortholog found through similarity searches. Interestingly, six miRNAs were found to be encoded by the genomes of all taxa analyzed. These were mir-10, mir-100, mir-1000, mir-315, mir-8 and mir-iab-4. Of those, mir-10 and mir-100 have been found in a large variety of species [87], while the remaining four have been found, with one exception, only in insects. Most of these miRNA species influence developmental pathways. Mir-iab-4 and mir-10, for example, are involved in the regulation of Hox genes, miR-100 has a role in apoptotic pathways, mir315 and mir-8 are implicated in the Wingless (Wg) signaling pathway, the latter being also involved in the regulation of neurogenic signals and gliogenesis in *Drosophila*. When comparing the genomes of the members of the *Anopheles gambiae* species complex, we identified an additional 21 genes that were common to all species in the complex (let-7, mir-1, mir-125, mir-1891, mir-190, mir-219, mir-252, mir-263a, mir-263b, mir-2765, mir-282, mir-283, mir-286, mir-305, mir-34, mir-7, mir-927, mir-929, mir-932, mir-993, mir-9b).

Table 4 also shows that, in most cases, the miRNAs identified have been annotated as such in Rfam. A significant number of putative miRNAs identified here, though, remained anonymous (percentage of the anonymous miRNAs varies from 0% – 48% depending on the species). Those miRNAs have been analyzed further and we found that the majority has, been annotated as miRNAs in RefSeq (again percentages varying from 67% - 100%). The remaining ones are simply classified as ncRNAs in RefSeq [88] without further details; we believe these to be miRNAs that are identified as such here for the first time. Their percentage is always smaller that 10% of the miRNAs accepted in our final set. Driven by the fact that more miRNAs are present in the very recent RefSeq database, we re-annotated our RNAz results versus RefSeq. In six genomes (*A. culicifacies, A.dirus, A. epiroticus, A. funestus, A. merus and A. stephensi)* an additional putative miRNA gene similar to mir-2796 was identified. In cases in which VectorBase listed annotated miRNA genes, between 32 and 73% of the ones identified in the present study were in common. In contrast, between 12.5% and 67% of the genes that we predict were not picked up by the VectorBase pipeline.

While writing this manuscript a study describing a transcriptome-wide analysis of miRNA expression in *A. gambiae* was published reporting previously unidentified miRNAs [85]. Although an overlap exists between “our” presumed miRNA genes and the ones reported there, there is a high inconsistency as far as total numbers of miRNAs identified in *A. gambiae* are concerned (see also miRNAs present in VectorBase). We stress that we have used a conservative approach in naming miRNA genes since we only made use of a bioinformatics pipeline. In all other anopheline species, the numbers of genes identified are about “equal”, still the actual identity overlap between them is, again, ~50%. This is clearly a result of the fact that pure computational approaches were used. Which values are “more correct” can only be evaluated in the future when users look at the data and determine by themselves, especially performing “wet experiments”.

### The snRNA genes

Without attempting a systematic approach, a by-product of searching for miRNA genes through the usage of RNAz was the identification of a series of additional non-coding RNA genes. The results are summarized in Table 5 and shown in Additional file 6. Genes coding for U1 spliceosomal RNA [89] were found most often; only in *A. minimus* was no gene identified. In addition to U1 RNA genes, genes for another 6 classes were discovered, namely for U4, U4atac, U5, U6atac, U11 and U12 RNAs (see [90]).

**Table 5:** **snRNA genes in anophelines**

	U1	U4	U4atac	U5	U6atac	U11	U12
*Albimanus*	3	2					
*Arabiensis*	5	1		1			1
*Atroparvus*	6	2		2		1	
*Christyi*	2	1				1	1
*Culicifacies*	1	1				1	1
*Darlingi*	4		1			1	
*Dirus*	4	1	1				
*Epiroticus*	5				1		
*Farauti*	4	2					
*Funestus*	2	1				1	1
*Gambiae* PEST	2	1			1		
*Gambiae* S	3	1	1			1	
*Maculatus*	1						
*Melas*	2						
*Merus*	4	1					1
*Minimus*		1					
*Quadriannulatus*	3	1					1
*Sinensis*	4	1				1	
*Stephensi*	1	1				1	

The Table lists the number and species of snRNAs identified in 19 anopheline taxa examined.

## Conclusions

This report presents a comprehensive search and scan of NGS genomic alignments to identify and annotate several families of non-protein encoding RNA genes. This work was an integral part of the project carried out by the “Anopheles Genomes Cluster Consortium” producing, using NGS, assemblied genomes for a collection of 19 anopheline species. Of all ncRNA families, we concentrated on those presented here, for different reasons, the most crucial of which being the possibility to obtain conclusive answers exclusively through the usage of computational methods. Thus, families such as Piwi-interacting RNAs [91] that, additionally, require a PCR approach for their identification were not part of the present study.

We succeeded in identifying and producing a preliminary computational analysis of a variety of genes. It is obvious, given the fact that only computational methods were used, that we chose a rather conservative approach in our analysis. Although this decision may lead to a relative lack of information, we think that this will be more helpful to the Anopheles research community; it is provided with a repertoire of genes that can function as molecular tools in a series of experimental designs, most prominently, but clearly not exclusively, for evolutionary studies at different levels. In addition to that, the study provided answers to some questions that had remained unanswered for several years. For example, it was clearly established that the presumed contiguous structure of the 5.8S rRNA in anophelines is a fact, in contrast to what is true for other insects, including *D. melanogaster*.

This study also demonstrated some weak points that are linked to the usage of NGS approaches in WGS of genomes. These pitfalls mostly affect the study of highly repeated segments such as, in our case, the ribosomal genes. Not only did we find that, in many of the genes examined, rDNA segments are entirely missing from the assemblies, we also noticed the presence of several mistakes in these assemblies. This remains a problem that will have to be solved, in the future, through the acquisition of longer sequence reads [92] or the development of enhanced software. Nevertheless, it is clear that significant conclusions can already be drawn, especially when one considers the availability of RNAseq experiments that accompany the whole genome sequencing. In our case, the availability of special RNAseq sets (*e.g.* designed for short RNA sequences), would have greatly improved several aspects of this study.

In conclusion we can say that the approach chosen by the “Anopheles Genomes Cluster Consortium” can be called successful. Joining a number of expert groups in the analysis of a large set of anopheline species to analyze the results obtained from the NGS-based genome analysis can clearly lead to the acquisition of a wealth of biological data, even considering some drawbacks due to the technologies available today.

### Availability of supporting data

The data sets supporting the results of this article are included within the article and its additional files. All the genomic assemblies and sequences used in this analysis are available at VectorBase (http://www.vectorbase.org). Consensus sequences described, and genes identified here have been submitted to VectorBase for inclusion in the corresponding species pages. Phylogenetic data have been submitted at TreeBASE (http://purl.org/phylo/treebase/phylows/study/TB2:S16748).

## Electronic supplementary material

Additional file 1: **List of the rDNA segments species used in the phylogenetic analyses.** The numbers indicate the corresponding segment in Table 1 or, in the case of *D. melanogaster*, the sequence stored with the accession number M21017. The accession numbers for the mitochondrial Cytochrome c oxidase subunit I (COI) and the mitochondrial 16 S rDNA (16S) refer to the corresponding accession numbers in the Genbank/EMBL databases. (-: not used). (DOCX 19 KB)

Additional file 2: **rDNA repeat sequences for the anophelines listed in Table** 2 **.** This txt file is essentially a FASTA-formatted file that contains all sequences determined and listed in Table 1. The sequences are listed alphabetically by species name. Where a sequence is missing, a dash (-) indicates this and, if known, the approximate length of the segment missing is indicated in the sequence description. Otherwise, the coordinates for each rDNA segment are given after the name of the segment (- is always counted as 1 nucleotide in the calculation of lengths in the file). (TXT 78 KB)

Additional file 3: **snoRNA genes identified in the anopheline genomes.** This txt file is essentially a FASTA-formatted file that contains all sequences determined and listed in Table 2. The sequences are listed alphabetically following the names of the taxa. The coordinates of the scaffold in which the sequence was detected iare indicated in the description line, followed by a short description of the computed stem structure (see Table 2 and Results) as well as, finally, the target sequence used. (TXT 630 KB)

Additional file 4: **tRNA genes identified in the anopheline genomes.** This txt file is essentially a FASTA-formatted file that contains all sequences determined and listed in Table 3. The sequences are listed alphabetically following the names of the taxa. The coordinates of the scaffold in which the sequence was detected are indicated in the description line, followed by a short description of the tRNA encoded. (TXT 1019 KB)

Additional file 5: **miRNA genes identified in the anopheline genomes.** This txt file is essentially a FASTA-formatted file that contains all sequences determined and listed in Table 4. The sequences are listed alphabetically following the names of the taxa. The coordinates of the scaffold in which the sequence was detected as well as the direction of transcription are indicated in the header line. The header also contains, when available, the coordinates and the sequence of the mature miRNA. (TXT 292 KB)

Additional file 6: **snRNA genes identified.** The table lists the species, the scaffold coordinates and the kind of snRNA identified. (TXT 17 KB)
