# Supplementary material for: Non-coding RNA gene families in the genomes of anopheline mosquitoes
Source: BMC Genomics. 2014 Nov 28;15(1):1038. doi: 10.1186/1471-2164-15-1038 (PMC4300560; doi:10.1186/1471-2164-15-1038)
Supplement: Supplementary file 1 — Additional file 1: List of the rDNA segments species used in the phylogenetic analyses. The numbers indicate the corresponding segment in Table 1 or, in the case of D. melanogaster, the sequence stored with the accession number M21017. The accession numbers for the mitochondrial Cytochrome c oxidase subunit I (COI) and the mitochondrial 16 S rDNA (16S) refer to the corresponding accession numbers in the Genbank/EMBL databases. (-: not used). (DOCX 19 KB) [file 12864_2014_6849_MOESM1_ESM.docx]

| **Species** | **Gene fragments** | | | | |
| --- | --- | --- | --- | --- | --- |
|  | **5.8S rRNA** | **18SrRNA** | **28SrRNA** | **COI** | **16S** |
| *A. albimanus* | 1-120 | 27-644, 927-1480, 1857-1965 | 4457-4602, 5233-5322, 6629-7070, 7762-7879 | HM030907 | - |
| *A. arabiensis* | 1-120 | 27-644, 927-1480, 1857-1965 | 3179-3324, 3943-4032 | DQ465294 | - |
| *A. atroparvus* | 1-120 | 27-644, 927-1480, 1857-1965 | - | - | - |
| *A. christyi* | 1-120 | 1857-1965 | - | - | - |
| *A. culicifacies* | 1-120 | - | 894-1038 | KF406660 | DQ202297 |
| *A. dirus* | 1-120 | 27-644 | 1736-2029 | AJ877449 | JX219732 |
| *A. epiroticus* | 1-120 | 27-644, 1857-1965 | - | AY789202 | - |
| *A. farauti* | 1-120 | 27-644, 927-1480, 1857-1965 | 3966-4109 | KF202472 | JX219741 |
| *A. funestus* | 1-120 | 27-644, 927-1480, 1857-1965 | 2997-3140, 3795-3883, 5200-5639 | JQ424685 | DQ287368 |
| *A. gambiae* | 1-120 | 27-644, 927-1480, 1857-1965 | 3211-3356, 3975-4064, 5357-5790, 6530-6655 | DQ465336 | NC_002084 |
| *A. maculatus* | 1-120 | 27-644, 927-1480, 1857-1965 | 2732-2821 | GQ259192 | DQ202298 |
| *A. melas* | 1-120 | - | 602-719 | DQ792580 | - |
| *A. merus* | 1-120 | 27-644, 1857-1965 | 2121-2238 | - | - |
| *A. minimus* | 1-120 | 27-644, 1857-1965 | 1874-2018 | GQ906996 | AY049016 |
| *A. quadriannulatus* | 1-120 | 27-644, 1857-1965 | 1676-1793 | DQ792581 | NC_000875 |
| *A. sinensis* | 1-120 | 27-644, 927-1480, 1857-1965 | 2701-2844, 3535-3624, 4937-5377, 6090-6207 | AB781786 | AF373609 |
| *A. stephensi* | 1-120 | 27-644, 927-1480, 1857-1965 | 3168-3311, 4002-4091, 5404-5844, 6557-6674 | AY877429 | AF034467 |
| *D. melanogaster* | M21017; 2722-2844 | M21017; 27-612, 871-1421, 1837-1942 | M21017; 3356-3701, 4259-4368, 5739-6177, 6808-6926 | HQ979116 | NC_001709 |
